# A4 Thoracolumbar Fracture Class Is Associated With a Greater Degree of Vertebral Height Loss in Conservatively Managed Patients

**DOI:** 10.7759/cureus.66402

**Published:** 2024-08-07

**Authors:** Ashwin Ghadiyaram, Asha Krishnakumar, Janan Leppo, Megan M Rajagopal, Nora T Poulos, Charles F Opalak, William C Broaddus, Brian M Cameron

**Affiliations:** 1 Department of Neurosurgery, Virginia Commonwealth University Health System, Richmond, USA; 2 Department of Internal Medicine, University of California San Diego, San Diego, USA; 3 Neurosurgery, Prisma Health Southeastern Neurosurgical and Spine Institute, Greenville, USA

**Keywords:** conservative management, spinal fracture, tlso brace, thoracolumbosacral orthosis, thoracolumbar fracture

## Abstract

Introduction: Thoracolumbar (TL) junction fractures are common, often resulting from high-energy trauma or osteoporosis, and may lead to neurological deficits, deformities, or chronic pain. Treatment decisions for neurologically intact patients remain controversial, with nonsurgical management often favored. The AO classification system has been used to characterize thoracolumbar fractures using fracture morphology and clinical factors affecting clinical decision-making for fracture management. This study aims to assess the radiographic outcomes of utilizing a thoracolumbosacral orthosis (TLSO) brace in neurologically intact patients with TL fractures based on the AO classification system.

Methods: A retrospective analysis of 43 patients was conducted using data from the VCU Spine Database on patients with TL fractures managed conservatively with a TLSO brace from 2010 to 2019. Demographic variables and radiographic measurements of anterior height loss were analyzed and stratified by AO fracture class.

Results: Significant differences were observed in anterior height loss between AO fracture classes, with A4 fractures showing significantly greater anterior height loss at initial presentation (27.6 + 4.8%) compared to A1/A2 (16.1 + 2.2%; p=0.049). At follow up, A4 fractures had a significantly greater anterior height loss (40.2 + 6.6%) than both the A1/A2 (22.4 + 2.9%; p=0.029) and A3 fracture classes (20.5 + 3.6; p=0.020).

Conclusions: The study highlights significant differences in anterior height loss among AO fracture classes, suggesting varying degrees of severity and potential implications for clinical management. While conservative treatment with TLSO braces may provide pain relief, surgical intervention may offer better structural recovery, especially in more severe fractures. Conservative management of TL fractures with TLSO braces may result in greater anterior height loss, particularly in A4 fractures, emphasizing the need for individualized treatment decisions. Further research, including prospective studies, is warranted to validate these findings and guide clinical practice effectively.

## Introduction

The thoracolumbar (TL) junction (T12-L1) and adjacent vertebrae in the spinal column ranging from T11 to L3 are the most common sites of spinal fractures due to the increased biomechanical stress at the transition from the rigid thoracic spine, with its associated sternum and ribs, to the mobile lumbar spine [1-3]. These injuries can occur in patients of all ages, and elderly patients are especially susceptible due to an age-related reduction in bone density as well as an increased incidence of osteoporosis [4].

The narrow spinal canal in the thoracic portion of the TL junction predisposes fractures in this region to compress or contuse the thoracic spinal cord, medullary cone, and nerve roots. Accordingly, 15-40% of TL fractures may have associated neurological deficits [5,6]. Severe trauma to the TL junction may also lead to paralysis or deformity, and lesser injuries can lead to limited daily activity due to chronic pain [7]. Appropriate management of these injuries is therefore essential for patient outcomes and quality of life.

The AO classification system is a classification system that has been established based on a consensus from the AO Spine Classification Group. The classification system has been used to characterize thoracolumbar fractures using fracture morphology and clinical factors important in clinical decision-making for fracture management [8].

There is ongoing controversy over the treatment of neurologically intact patients with thoracolumbar fractures. Generally, the treatment of neurologically intact patients is nonsurgical due to conservative management with early mobilization or bracing, leading to pain relief and improved functional outcomes [9]. A randomized controlled trial at the University of Toronto [10] studied the efficacy of bracing using a thoracolumbosacral orthosis (TLSO) versus early immobilization without bracing in patients who suffered AO Class A3 fractures at the T12-L2 levels. The study revealed that the clinical outcomes were similar in the brace and no-brace groups [10]. They also concluded that there were no significant differences in radiographic and clinical outcomes at the six-month follow-up [10]. Nataraj et al. conducted a retrospective cohort study looking at surgical versus nonsurgical management in patients with thoracolumbar burst fractures, and they found no significant differences in functional outcomes between patients treated surgically or conservatively at a six-month follow-up period [11].

Fracture stability is an important factor in determining the optimal treatment of thoracolumbar fractures. Conservative management for thoracolumbar fractures is performed based on physical examinations and imaging studies and should be considered first for managing mechanically and neurologically stable fractures [7]. Typical conservative treatment involves a TLSO brace for 8 to 12 weeks [7]. Operative intervention for thoracolumbar fractures is generally performed when there is neurological or mechanical instability, and the goal of operative treatment is decompression of the spinal canal and nerve roots for neurological recovery, restoration, and maintenance of vertebral height and spinal alignment [7].

While studies have investigated functional and radiographic outcomes in patients with A3 and A4 fracture classes, these studies have grouped the two classes, which limits conclusions that can be drawn on differences in outcomes between the two fracture classes [12,13].

This study aims to investigate the radiographic results of utilizing a TLSO brace in neurologically intact patients with various thoracolumbar fractures based on the AO classification system. We seek to provide evidence-based, quantitative insight into fracture progression following conservative management of a thoracolumbar junction fracture utilizing a TLSO. 

## Materials and methods

The VCU Spine Database was queried for patients who had thoracolumbar fractures that were conservatively managed with a TLSO brace between 2010 and 2019. The AOSpine Thoracolumbar Spine Injury Classification System was used to classify these patients’ injuries [14]. Additionally, due to the similarity of A1 and A2 fracture classes being non-comminuted fractures, A1 and A2 fracture classes were grouped for statistical analysis. Patient demographic variables, including sex, AO fracture class, fracture level, body mass index (BMI), height, smoking history, and days to follow-up, were summarized using means and standard deviations or frequencies and percentages.

Our institution’s protocol for management with a TLSO involves a shared decision-making process, where the risks and benefits are discussed with the patient, and the patient is then fitted for a TLSO brace. The patient is then counseled on how to proceed with the brace, which includes instructions to always wear the brace when the patient is out of bed and for the patient not to engage in strenuous activity or exercise. Generally, a patient would wear their TLSO for the whole day for the first 12 weeks after discharge and for half a day for an additional 3-4 weeks. This is consistent with other studies that have described the management of patients with TLSO [15-17]. For pain management, a regimen is established based on the patient’s pain on presentation, throughout their inpatient stay, and any chronic conditions they may have that can affect the patient’s pain assessment. On discharge, this regimen is usually a two-week prescription, or a duration based on the next follow-up visit to their primary care physician or specialty clinic, and can consist of an extended-release opioid, such as morphine, to be taken every 12 hours along with a short-acting opioid, such as oxycodone or hydromorphone, to be taken once every four hours as needed for severe pain. The patient would be scheduled for a follow-up visit at the neurosurgery clinic, and assessments are made based on how the patient’s pain is, and how they are performing their daily activities with the TLSO brace. When the patient can state that they are able to perform activities with little limitation with their brace, they are then allowed to arrange follow-up with neurosurgery on an as-needed basis.

**Figure 1:**
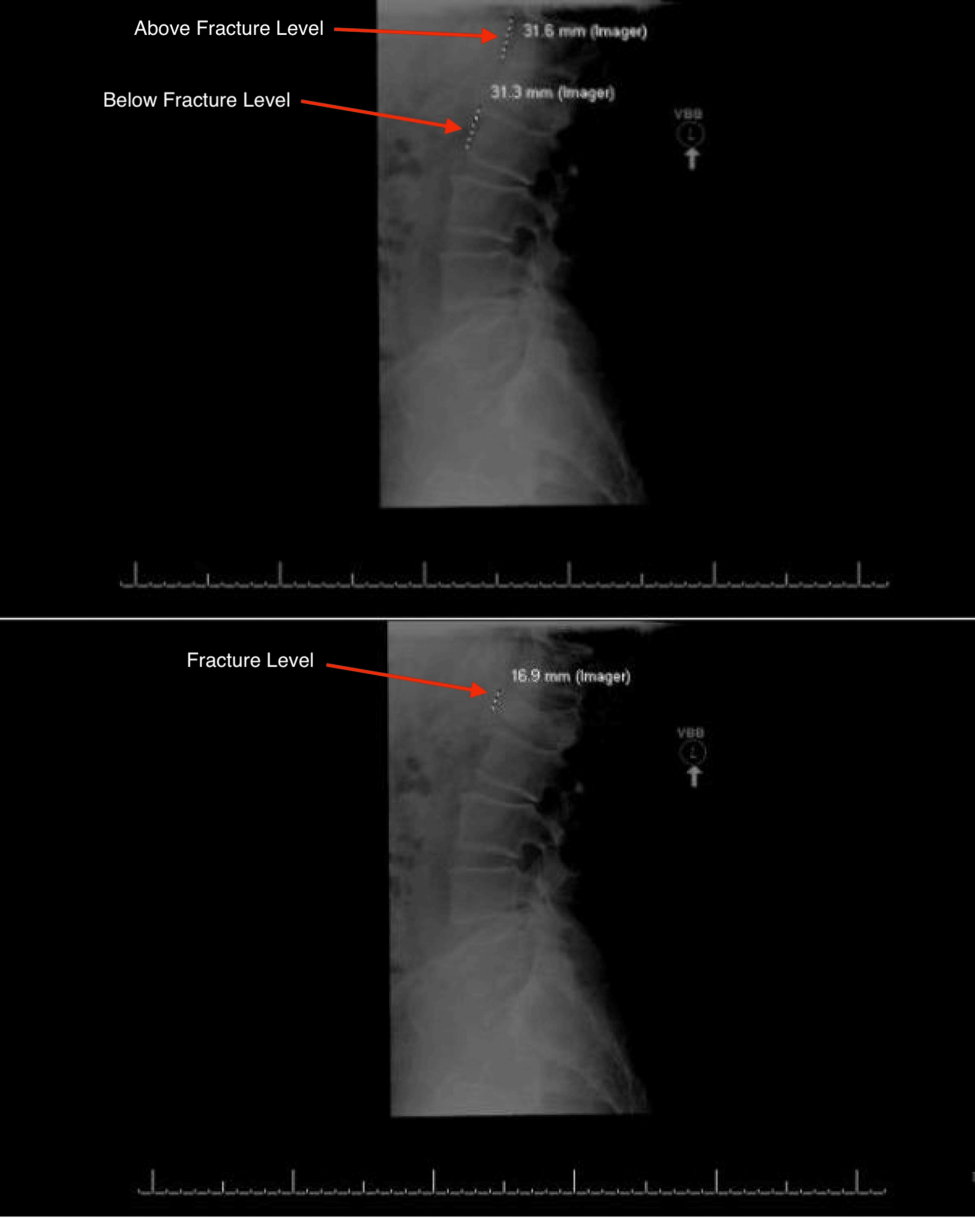
Estimated anterior height loss is based on vertebral body measurements above and below the fracture (top) and measured anterior height loss at the level of the fracture (bottom)

Radiographic measurements of anterior height loss of the vertebral body implicated in each given fracture were measured for each patient at their initial visit and average follow-up visit time. The method for anterior height loss measurement was determined radiographically by first approximating the height of the vertebral body by taking the height of the vertebral body above and below the fracture and taking the average. Next, the height of the fractured vertebral body was measured. The measured height was compared to the approximated vertebral body height, and the percent height loss was calculated as the final measure of anterior height loss in the form of a percent. This measurement method was performed for each patient on initial imaging at the time of fracture and follow-up after being conservatively managed with a TLSO brace. A radiographic view of the anterior height loss calculation, based on vertebral body measurements above, below, and at the level of the fracture, can be seen in Figure 1. The change in height loss between initial imaging and follow-up imaging was calculated as well to determine the change in anterior height loss over time in the form of a percent. 

**Figure 2:**
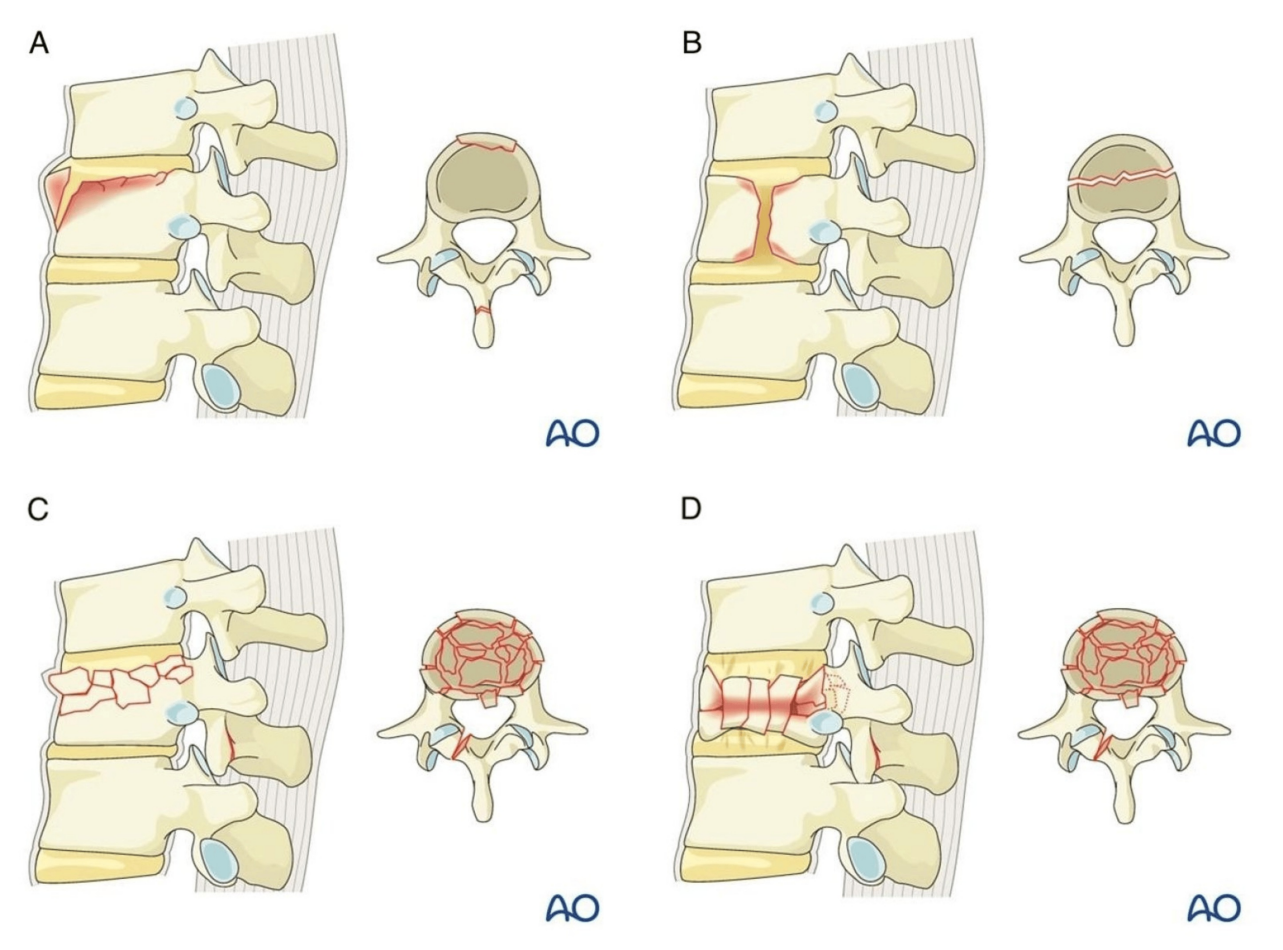
Visual depiction of the AO fracture classes A: A1 Fracture Class characterizing wedge compression fractures involving a single endplate of the vertebral body without involving posterior wall. B: A2 Fracture Class characterizing split or pincer fractures involving both endplates of the vertebral body without involving the posterior wall. C: A3 Fracture Class characterizing incomplete burst fractures involving a single endplate of the vertebral body along with involving the posterior wall. D: A4 Fracture Class characterizing complete burst fractures involving both endplates of the vertebral body along with the posterior wall [19].

**Figure 3:**
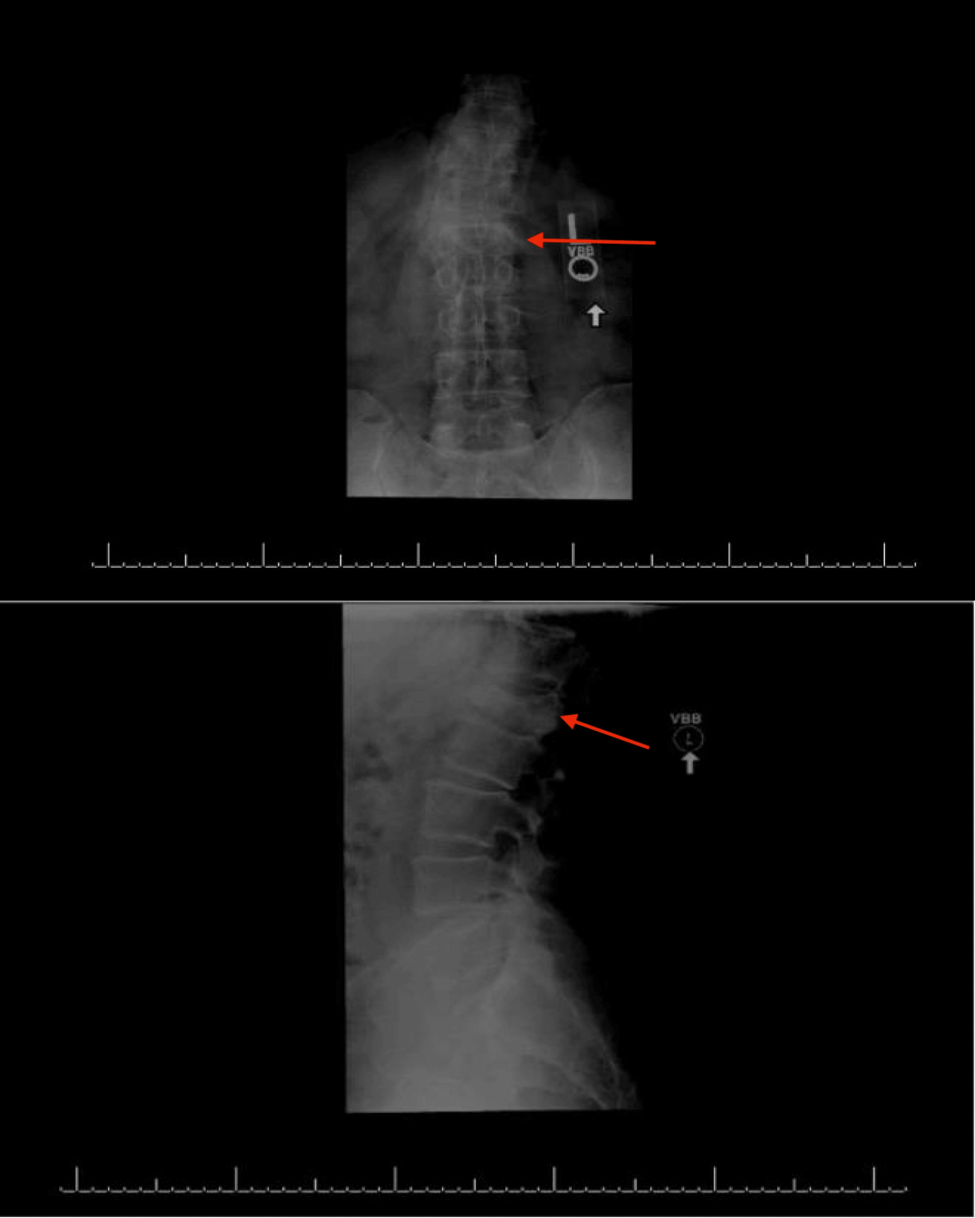
Anterior-posterior view of an A4 fracture (top) and sagittal view of an A4 fracture (top), with the red arrows pointing to the fracture level

The AO Classification System is a spinal-fracture classification system developed by the AOSpine Classification Group, a subgroup of the AOSpine Trauma Knowledge Forum consisting of an international group of academic spine surgeons [14]. The AO Classification System is used to provide consistency in injury diagnosis and treatment, and the classification types analyzed in this study are shown in Figure 2. A1 fractures, also known as wedge or impaction fractures, refer to fractures of one or both endplates, but the fractures do not connect [14,18]. A2 fractures refer to fractures of the vertebral body in which the fractures involve both endplates, also known as split or pincer-type fractures [18]. A3 burst fractures are fractures of a single endplate and can involve a vertical fracture of the lamina [18]. A4 burst fractures involve both endplates and are a sign of compression forces [18]. Figure 3 shows anterior-posterior and sagittal views of an A4 fracture at our institution.

Statistical analysis

Patient data was stratified based on the AO fracture class for analysis. Anterior height loss outcomes were compared between cohorts, with continuous and categorical variables evaluated via the T-test and Fisher’s exact test, respectively. Continuous variables were presented as the mean + standard error of the mean. Statistical significance was set to a p-value < 0.05. Predictors of percentage change in anterior height loss were identified via standard multivariate linear regression. Statistical analysis was performed using R (Version 4.1.0).

## Results

A total of 43 patients were included in this study, with patient demographic variables summarized in Table 1. Outcomes of anterior height loss in all AO fracture classes are summarized in Table 2. Other outcomes of TL fracture characteristics, including posterior height loss, vertebral-body angulation, segmental kyphosis, and thoracolumbar junctional kyphosis are summarized in Table 3. Cohort comparisons between AO fracture classes are summarized in Table 4.

**Table 1:** Demographics of patients with a TLSO at our institution

Variable	Categories	Value (Range)	%
Number of patients		43	100
Sex			
	Male	27	62.8
	Female	16	37.2
Age (years)		48.3 + 18.0 (21-86)	
	≤65	36	83.7
	>65	7	16.3
Body Mass Index		26.1 + 4.9 (18.9-40.8)	
	≤30	37	86.0
	>30	6	14.0
Height (centimeters)		171.8 + 11.6 (152-188)	
Tobacco Use?			
	Yes	16	37.2
	No	27	62.8
Fracture Level			
	T11	1	2.3
	T12	11	25.6
	L1	24	55.8
	L2	5	11.6
	L3	2	4.7
Fracture Type			
	Non-Comminuted	22	51.2
	Comminuted	21	48.8
AO Fracture Class			
	A1	18	41.9
	A2	4	9.3
	A3	11	25.6
	A4	10	23.3
History of Osteoporosis			
	Yes	2	4.7
	No	41	95.3
Follow-Up (days)		49.9 + 22.8 (16-110)	

**Table 2:** Anterior height loss at the time of fracture, as compared to follow-up in all three fracture classes

Fracture Class	Number of patients	Anterior Height Loss (%) at time of fracture	Anterior Height Loss (%) at follow-up	Percent change in Anterior Height Loss (%)
A1/A2	22	16.1 + 2.2	22.4 + 2.9	62.7 + 22.7
A3	11	18.0 + 2.9	20.5 + 3.6	31.0 + 19.8
A4	10	27.6 + 4.8	40.2 + 6.6	56.4 + 15.5
TOTAL	43	19.2 + 1.8	26.1 + 2.6	53.1 + 13.1

**Table 3:** Comparison of thoracolumbar fracture characteristics at the time of fracture and at follow-up by fracture class

Variable	Fracture Class	Mean ± SD at the Time of Fracture	Mean ± SD at Follow-Up
Posterior Height Loss (%)			
	A1	3.5 ± 2.9	6.1 ± 3.8
	A2	4.6 ± 4.4	9.1 ± 6.8
	A3	4.4 ± 3.3	9.4 ± 9.9
	A4	13.6 ± 14.0	20.5 ± 23.9
Vertebral Body Angulation (degrees)			
	A1	9.9 ± 4.1	13.8 ± 4.8
	A2	4.5 ± 3.4	9.3 ± 6.3
	A3	8.3 ± 4.9	14.2 ± 7.4
	A4	7.9 ± 4.8	11.6 ± 5.8
Segmental Kyphosis (degrees)			
	A1	8.6 ± 5.7	11.6 ± 6.8
	A2	5.0 ± 2.2	8.8 ± 4.5
	A3	5.1 ± 4.7	9.7 ± 5.5
	A4	8.9 ± 6.5	13.5 ± 8.7
Thoracolumbar Junctional Kyphosis (degrees)			
	A1	5.2 ± 4.7	6.6 ± 6.1
	A2	2.8 ± 1.7	6.8 ± 4.3
	A3	6.3 ± 6.2	5.6 ± 6.0
	A4	4.2 ± 3.5	6.5 ± 5.0

**Table 4:** p-values of cohort comparisons of anterior height loss at the time of fracture versus at follow-up

Cohort Comparison	Anterior Height Loss at time of fracture (p-value)	Anterior Height Loss (p-value)	Percent change in Anterior Height loss (p-value)
A1/A2 vs. A3	0.59	0.68	0.30
A1/A2 vs. A4	0.049	0.029	0.82
A3 vs. A4	0.11	0.020	0.33

The A4 fracture class exhibited significantly greater anterior height loss at the time of fracture than the A1/A2 fracture class (A1/A2: 16.0 + 2.2, A4: 27.6 + 4.8, p = 0.049). Additionally, the A4 fracture class also exhibited significantly greater anterior height loss at follow-up in comparison to the A1/A2 fracture class (A1/A2: 22.4 + 2.9, A4: 40.2 + 6.6, p = 0.029). Moreover, the A4 fracture class exhibited significantly greater anterior height loss at follow-up than the A3 fracture class (A3: 20.5 + 3.6, A4: 40.2 + 6.6, p = 0.020). There was no statistically significant difference between the degree of anterior height loss at the time of fracture between the A3 and A4 fracture classes. Additionally, there were no statistically significant differences in percent change in height loss over time among the fracture classes. Furthermore, there were no statistically significant differences between the A1/A2 fracture classes and the A3 fracture classes in any measured anterior height loss metrics.

Multivariate linear regression in determining predictors of percent change in anterior height loss was characterized by a statistically significant contribution of the L3 fracture level (p = 0.025) and the anterior height loss at the time of fracture (p = 0.0039), as summarized in Table 5. There were no significant contributions from AO fracture class or fracture levels other than L3 fracture level, age, gender, BMI, or smoking status.

**Table 5:** Multivariate linear regression for percent change in anterior height loss at follow-up

Variable	Coefficient	Standard Error	p-value
Intercept	46.65	93.06	0.62
A2 fracture class	69.98	55.75	0.22
A3 fracture class	-10.42	30.75	0.74
A4 fracture class	-10.99	35.94	0.76
L2 fracture level	-55.25	40.17	0.18
L3 fracture level	139.65	58.99	0.025
T11 fracture level	96.01	83.82	0.26
T12 fracture level	49.60	28.13	0.088
Age	0.73	0.71	0.32
Gender	12.40	25.10	0.62
Body Mass Index	1.21	2.81	0.67
Smoking Status	-41.56	29.77	0.17
Anterior Height Loss at the Time of Fracture	-3.41	1.09	0.0039

## Discussion

In this retrospective case series, there were significant differences in degrees of anterior height loss when making cohort comparisons between the A1/A2, A3, and A4 thoracolumbar fracture classes. The A4 fracture class exhibited a significantly greater degree of height loss than the A1/A2 class at the time of fracture. Additionally, the A4 fracture class exhibited substantially greater height loss than the A1, A2, and A3 classes at follow-up. These findings can be explained by the increasing severity of fracture morphology within the AO class. Specifically, A1/A2 fractures are non-comminuted fractures, whereas A3 and A4 fractures are comminuted fractures, such that the A4 class involves a complete burst of the vertebral body while the A3 class does not. Aboudu et al. studied two randomized controlled trials that studied surgical vs. non-surgical interventions in thoracolumbar burst fractures, and they found that the degree of kyphosis was reduced during follow-up in surgically treated patients versus increased degrees of kyphosis in the non-operatively managed patients [20]. However, pain scores and time to return to work had no significant differences between the two groups [20]. Another study performed by Mahmoodkhani et al. explored a non-randomized clinical trial looking at patients with fractures at the thoracolumbar junction, classified using the Thoracolumbar Injury Classification and Severity Scale. The study concluded that since surgery is often indicated in severe thoracolumbar fracture morphology, the surgical outcomes in the study showed that surgical intervention can benefit these patients [21], who underwent a standardized long fusion protocol, with outcome measures being fusion rate, hardware imbalance, sagittal imbalance, and functional outcomes [21]. They found that for patients with a TLICS >4, which is considered the score threshold for surgical intervention, long fusion was effective in correcting TL junctional kyphosis, maintaining vertebral height, and restoring functional and neurologic outcomes in patients with thoracolumbar fractures [21]. The study concluded that since surgery is often indicated in severe thoracolumbar fracture morphology, the surgical outcomes in the study showed that surgical intervention can benefit these patients [21].

While surgery is indicated in fractures with more severe morphologies, such as burst fractures, as compared to less severe wedge fractures, conservative management is often appropriate for neurologically intact patients [22]. Krompinger et al. pointed out that factors that consist of fracture stability, degree of canal compromise, and patient evaluation need to be taken into consideration before deciding whether a patient receives surgery or conservative management for treatment [22]. For patients without neurological deficits, nonoperative management can allow for a successful rehabilitation and functional recovery course [22].

Studies have also explored the benefits of operative management in neurologically intact patients. A comparative study by Denis et al. investigated operative versus nonoperative management in patients with thoracolumbar fractures who were neurologically intact [23]. They found that all patients who underwent surgery without unrelated disability were able to return to work, whereas only 25% of patients receiving conservative management were able to return to work [23]. Additionally, 17% of the patients in the nonoperative group developed neurological deficits, and the study concluded that prophylactic operative management may be beneficial for neurologically intact patients with a TL fracture [23]. This idea was further explored in a meta-analysis by Gnananenthiran et al., and they found that neurologically intact patients undergoing surgery versus nonoperative management had some improvement in kyphosis but no significant differences in improving pain or function at an average of four years after injury [1]. Additionally, the group undergoing surgery had more complications, such as infection, hardware-related complications, and iatrogenic injury, along with a higher cost [1]. 

Our present study looked at neurologically intact patients. The literature showed that surgery may provide a benefit for patients without neurological deficits but that conservative management may also be appropriate. Considerations of fracture morphology, comorbidities, and the degree of vertebral height loss can be important factors in deciding whether a patient may benefit from TLSO bracing or surgery and can apply to patients in this cohort. The primary outcome in this retrospective study was the percent change in anterior height loss, which indicated that this study was a study of radiographic outcomes rather than outcomes in patient quality of life. Outcome measures for QOL have included the Visual Analogue Scale (VAS), the Oswestry Disability Index (ODI), and the 36-item Short Form Survey (SF-36). The Visual Analogue Scale (VAS), which is a subjective outcome measure looking at the severity of pain and the extent to which the injury disrupts the patient's life and comparing both preinjury and postinjury scores, may be able to study outcomes in TL spinal injuries further [24]. Another outcome measure of QOL that can be used in studying recovery after spinal fractures is the Oswestry Disability Index (ODI), which is a questionnaire looking at pain severity and the extent to which pain medication can help in managing pain from fractures [25].

Suzuki et al. delved into measures of QOL in patients with thoracic and lumbar fractures. They studied QOL measures and activities of daily living (ADL) in patients with osteoporotic fractures at the thoracic and lumbar levels [26]. They concluded that initial severe fracture deformation with crush fractures, as compared to endplate fractures, is the worst prognostic factor for severe chronic pain, disability, and deterioration of ADL and QOL [26]. These findings indicate that fracture morphology can predict quality of life and that, regarding the AO classification system, compression, and crush fractures (A3/A4) can indicate a negative prognostic indicator for quality of life as compared to fractures with a lesser extent of morphological injury (A1/A2) [26]. Another study by Rezvani et al. studied two surgical techniques for spinal ligamentotaxis at multiple spinal levels, including the TL level, and their outcome measures included the postoperative VAS score, degree of retropulsion correction, mid-sagittal anterioposterior (AP) diameter (MSD), postoperative kyphotic deformity correction, and anterior height of the fractured vertebra [27]. They concluded that the postoperative VAS score was significantly lower in patients receiving in-fracture pedicular case screw insertion versus patients in the control group not receiving an in-fracture pedicular screw insertion (p = 0.004), and other quantitative outcome measures also backed this finding that in-fracture pedicular screw insertion may be safe and effective in correcting the structural deficits sustained from spinal ligamentotaxis [27]. Thus, VAS may be an effective outcome measure for studies looking at TL spinal fractures.

Wood et al. [28], in a retrospective cohort study, found that patients with A3 and A4 fractures had similar ratings of functionality and pain, using the ODI and SF-36 questionnaires, when undergoing operative versus conservative management [28]. Another study by Jung et al. found that patients with recovered compression fractures with non-surgical management may still exhibit a decline in functional and pain outcomes after a 12-month follow-up [29]. Wood et al. followed up their original retrospective study with a prospective study that showed that after a long-term follow-up of 16 years, patients with a stable burst fracture who were treated nonoperatively exhibited significantly improved pain and function compared to those who underwent surgery [30].

While our study showed that the A4 fracture class had a significant increase in anterior height loss despite TLSO bracing, our results also showed that A3 thoracolumbar fractures did not exhibit a significant difference in anterior height loss at follow-up. This finding may point to the stability of the fracture itself, as A3 burst fractures only involve one endplate, whereas A4 fractures involve both endplates and may point to the lack of stability of the fracture. Hillier et al. found that a greater height loss, especially in unstable fractures, may serve as a negative prognostic indicator in the recurrence of the spinal fracture or even the occurrence of non-spinal fractures such as hip fractures and a negative prognostic indicator for mortality [31]. This is in line with our study, showing that a more unstable fracture morphology (A4) incurs a larger anterior height loss at follow-up, and therefore, patients with A4 fractures have decreased radiographic recovery even with a brace.

Our multivariate analysis showed that the L3 fracture level was a significant predictor of increased change in percent anterior height loss (β coefficient = 139.65; p=0.025). This finding may point to the location of the L3 vertebra being in the middle of the lumbar spine. In studying the structural integrity of the spine, an important consideration has been that spinal pathology at both levels above and below L3 can affect the L3 lumbar segment and therefore contribute to chronic lumbar instability [2,32]. In our study, we noted that while patients with A4 fractures may be indicated for surgical management, characteristics such as patient choice, factors including comorbidities, age, and history of previous surgeries are taken into consideration to decide on surgical versus conservative management [1,22]. Additionally, the AO spine classification used in our present study does not provide a direct recommendation on whether a patient is indicated for surgery. However, the TLICS classification uses a scoring method that assigns a score based on fracture morphology, neurologic involvement, involvement of the cord and conus medullaris, and involvement of the posterior ligamentous complex, with a maximum score of 12 [33]. Patients with a score of 0-3 are recommended for nonoperative management, and patients with a score greater than 4 are recommended for surgical management, whereas patients with a score of 4 can be recommended for either treatment approach, based on the surgeon’s decision [33]. This scoring system may be useful for patients with more severe fractures and neurologic impairment and can better serve as a tool for recommending surgery [33].

A study by Withrow et al. studied the validation and comparison of TLICS, a modified TLICS (mTLICS), and the AO classification system in terms of the predictive accuracy of treatment recommendations, and they found that the mTLICS and TLICS had a higher specificity in terms of the predictive accuracy of treatment recommendations, but the AO classification system had a higher sensitivity in terms of predicting the accuracy of treatment recommendations [34]. The high specificity of the TLICS system can run into the risk of a false negative, while the recommendation for conservative management for patients who might need more aggressive treatment can run the risk of developing neurological deficits due to the undertreatment [34]. However, the high sensitivity of the AO classification system may result in false positives, where patients who can benefit from conservative management are recommended aggressive treatment, which can result in surgical complications and a higher healthcare cost [34].

Surgical recommendations based on the fracture class of the AO classification system in the study by Withrow et al. show that the AO classification system may be better used for patients who are more suited for conservative management. In our present study, since all patients in our cohort were neurologically intact, the TLICS system may not be as effective for specific presentations [34]. Therefore, the AO classification was used as these patients were better suited for recommendations for conservative management, and the characteristics of the patient’s presentation were taken into consideration for the recommendation to conservatively manage their TL fractures in all four fracture classes.

Spinal stability relies on how each segment fits into the shape of the spinal column, with the alignment of the lumbar spine being characterized by lordosis [35]. Stabilization of the unstable injured motion segments is important in preventing further injury [2], and our study shows that while the A3 burst fracture may inherently be more stable with or without a TLSO to mitigate further anterior height loss, the clinically significant radiographic progression of A4 thoracolumbar fractures without neurologic injury despite TLSO bracing may represent a subset of patients that may benefit from early surgical intervention.

Limitations

This retrospective case series has limitations. First, the sample size was small, and each patient had a different mechanism for their injuries, making it difficult to account for the exact mechanism of injury. Additionally, comorbidities such as osteoporosis and osteopenia, metabolic disorders such as hyperparathyroidism, and nutritional deficiencies could affect recovery from fractures [36,37]. Having a larger sample size, along with controlling for comorbidities and mechanisms of injury, could yield additional findings beyond those identified in this study and could be applied to clinical practice in managing thoracolumbar fractures.

The outcome measures were also determined by a quantifiable physical measure and not patient-to-patient subjective characteristics, which could have influenced the data from a patient QOL perspective. This study only looked at the percent change in anterior height loss, which made it a study of objective measures rather than subjective measures of QOL. While adding QOL indicators to this study may have skewed results based on subjective patient indicators, it can add to the study by looking at how conservative management can affect QOL in different fracture morphologies.

## Conclusions

This retrospective cohort study studied the radiographic outcomes of the use of thoracolumbosacral orthoses (TLSO) in patients with thoracolumbar fractures. Our study shows that conservative management of the A4 fracture class is associated with a greater degree of anterior height loss at follow-up in comparison to less severe fractures in the AO class system. The findings from this study can be due to the instability of the fracture type playing a role in recurrent pathology and how these fractures can affect adjacent vertebral segments and spinal alignment. These findings can assist in decision-making for patients with thoracolumbar fractions who are candidates for management with TLSO braces.

Future research will focus on prospective, controlled studies of radiographic and functional outcomes using imaging and indicators of quality of life in patients treated conservatively and surgically for thoracolumbar fractures, with specific attention to A4-type fractures.
